# Nighttime Light Hurts Mammalian Physiology: What Diurnal Rodent Models Are Telling Us

**DOI:** 10.3390/clockssleep3020014

**Published:** 2021-04-01

**Authors:** Jorge Mendoza

**Affiliations:** Institute of Cellular and Integrative Neuroscience CNRS UPR3212, University of Strasburg, 8 allée du Général Rouvillois, 67000 Strasbourg, France; jmendoza@inci-cnrs.unistra.fr; Tel.: +33-03-8845-6696

**Keywords:** light pollution, diurnal, circadian, sleep, mood, metabolism, human, *Arvicanthis*

## Abstract

Natural sunlight permits organisms to synchronize their physiology to the external world. However, in current times, natural sunlight has been replaced by artificial light in both day and nighttime. While in the daytime, indoor artificial light is of lower intensity than natural sunlight, leading to a weak entrainment signal for our internal biological clock, at night the exposure to artificial light perturbs the body clock and sleep. Although electric light at night allows us “to live in darkness”, our current lifestyle facilitates nighttime exposure to light by the use, or abuse, of electronic devices (e.g., smartphones). The chronic exposure to light at nighttime has been correlated to mood alterations, metabolic dysfunctions, and poor cognition. To decipher the brain mechanisms underlying these alterations, fundamental research has been conducted using animal models, principally of nocturnal nature (e.g., mice). Nevertheless, because of the diurnal nature of human physiology, it is also important to find and propose diurnal animal models for the study of the light effects in circadian biology. The present review provides an overview of the effects of light at nighttime on physiology and behavior in diurnal mammals, including humans. Knowing how the brain reacts to artificial light exposure, using diurnal rodent models, is fundamental for the development of new strategies in human health based in circadian biology.

## 1. Introduction

Earth’s 24 h rotation induces cycles of day and night that synchronize the physiology of living organisms [1]. Thus, sunlight is the most important natural signal to entrain our rhythms of sleep, feeding, body temperature, and metabolism. Moreover, light effects on physiology are modulated by seasons, when the length and intensity of natural light change. Therefore, sunlight impacts daily and seasonal physiology [2].

In the beginning, organisms (including humans) regulated their daily activities according to the natural dawn and dusk. Then, with the industrial revolution, a relevant event in the human history, several changes arrived to “facilitate” human life; among them the discovery of electric light. In the last part of the industrial revolution (1879), Thomas A. Edison invented the electric light bulb that was immediately used for both domestic and industrial tasks. It is very likely that at that time people did not visualize what would be the impact of this event in biology and health. Today, it is well known that artificial light affects the physiology of living organisms, and mainly the biological functions with rhythmic properties.

One of principal effects of artificial light at night on physiology is the suppression of the hormone melatonin (MEL); this indicates that light regulates the neuroendocrine system [3]. Notwithstanding, because MEL is rhythmically secreted at night under the control of the body clock, light also affects the circadian system. Furthermore, since retina projections target directly to other brain regions, beyond the circadian clock, light (artificial or natural) may also influence and regulate functions such as mood, cognition, and metabolism [4]. The study of the circadian and non-circadian mechanisms underlying the effects of light on physiology is essential for the understanding of when and how light is beneficial or detrimental for health.

## 2. The Core of the Body Clock Entrained by Light: Following the Right Path

To recognize the endogenous structure able to tick every function in our body and to synchronize to day-night cycles, the first studies identified the anatomical pathway by which light enters to the body and entrains circadian rhythms. Therefore, one of the first experimental approach was to track pathways and brain targets using tracers injected into the eye. Hence, in rats, the retino-hypothalamic tract (RHT) was characterized as the principal monosynaptic projection from the retina to the ventral hypothalamus [5]. Interestingly, this study had already indicated that the RHT arises from retinal ganglion cells, which years later were identified as melanopsin-containing cells [6]. Then, two studies revealed that the ablation of this retino-recipient area in the ventral hypothalamus, harboring the suprachismatic nucleus (SCN), eliminates behavioral and hormonal rhythms in rats [7,8]; these studies gave the first recognition to the SCN as the central circadian clock in mammals [9].

The SCN is a bilateral mid-nucleus in the anterior ventral hypothalamus with approximately 10,000 neurons per nuclei. It contains a diverse amount of peptides and neurotransmitters, all of them with specific functions on the reception, integration and transmission of biological timing [9,10]. The inhibitory neurotransmitter gamma-Aminobutyric acid (GABA) is contained in almost every cell in the SCN. GABA production and release show a daily and circadian rhythm with a main activity in the middle of daytime. GABA has an important role in the coupling of clock cells into the SCN [11].

Anatomically, the SCN is subdivided into a ventral region containing vasointestinal polypeptide cells (VIP) and a dorsal vassopresinergic (AVP) region. Whereas VIP cells are the gate to transmit light information from the retina to the dorsal SCN clock [12,13], AVP neurons are the clock core that integrate this information. The circadian time is then transmitted to the rest of the brain and body to entrain peripheral clocks and physiological rhythms [14,15]. Moreover, VIP clock cells (as GABA) play a role in the SCN clock coupling; when VIP or its receptor VPAC2 are deficient, behavioral and molecular rhythms are disrupted in mice [12]. Importantly, recent studies reveal a new and interesting role of VIP neurons not only in the reception of light and SCN cell coupling, but also in the control of sleep at night in mice [16]. This gives a diverse and dynamic role of each subgroup of clock cells into the SCN.

Research on the molecular mechanisms of circadian oscillations in the SCN has advanced considerably [17]. The basis of these mechanisms depend on two principal feedback loops (a negative and a positive one) of transcription and translation of genes and proteins. The two transcription factors, *Clock* and *Bmal1*, once translated into proteins, form a dimer complex with the capacity to go back to the nucleus and bind at the site of transcription for genes such as *Period* (*Per1-3*) and *Cryptochrome* (*Cry1-2*) at the middle of the day. At the beginning of the night, the protein products PER and CRY also have the ability to form dimers to return to the nucleus and inhibit the CLOCK/BMAL1 activity, and therefore their own gene transcription [18]. To inform the other brain regions and organs in the body of the circadian time, the molecular clockwork uses the named clock-controlled genes (CCG), such as AVP, VIP, or the humoral factors Prokinotecin 2 (PK2) or the transforming growth factor alpha (TGF-alpha) [19].

## 3. The Retina-Brain Network: The Route to Synchronize (or Desynchronize) Circadian Rhythms and to Regulate Behavior by Light

In the retina, cells from the ganglion layer contain the photopigment melanopsin. These intrinsically photosensitive retinal ganglion cells (ipRGCs) have a maximum sensitivity to blue short-wavelength light (480 nm), and play a fundamental role for non-image-forming visual functions [20]. In rodents (mice), five subtypes (M1-5) of ipRGCs have been characterized [21]. Interestingly, in a recent study, the function of human ipRGCs was evaluated and at least three subtypes of ipRGCs were revealed according to their electrical activity responses to light; these responses are similar between rodents and humans [22].

ipRGCs (M1 subtype) fire in a tonic manner after light stimulation, then relaying this electrical information to the SCN clock. In addition, in nocturnal mice, ipRGCs contact other retino-recipient brain regions beyond the SCN, such as the intergeniculate leaflet (IGL), the olivary pretectal nucleus (OPN), the peri-habenular region (PHb), and indirectly (via the IGL) the lateral habenula (LHb) [23,24,25]. Extra-SCN ipRGC’s projections have also been reported in the diurnal rodent *Arvicanthis niloticus* [26,27]. Furthermore, M1-type melanopsin cells are subdivided in two types: those containing the transcription factor *Brn3b* that project to the extra-SCN brain sites (e.g., midbrain, thalamus), and the *Brn3b*-negative cells that project to the SCN [24,28]. Extra-SCN projections allow light to influence a wide range of non-image forming beyond the photic entrainment of the clock (e.g., sleep, mood, and metabolism).

In the SCN, ipRGC’s axons from the RHT contact principally ventral VIP cells, which contain glutamate and PACAP (pituitary adenylate cyclase-activating peptide) receptors. When light stimulates ipRGCs at night, in the RHT terminals glutamate or PACAP are released to bind their receptors. This leads to an important increase of calcium (Ca^2+^) flux and the activation of specific intercellular cascades which include the protein kinase A (PKA), cAMP response element binding protein (CREB), and the CREB-regulated transcription coactivator 1 (CRTC1) [29]. When CREB is phosphorylated and partnered to CRTC1, it binds the CRE sequences in the promotor of *Per* (*Per1-2*) genes to induce their transcription. Importantly, the effects of light on the clock physiology are time-dependent. Light stimulation at early and late-night delays and advances, respectively, the clock and the over rhythms (e.g., locomotor activity, melatonin) [30,31].

## 4. Light Effects on Physiology: Differences between Diurnal and Nocturnal Species

In chronobiology, nocturnal species such as mice, rats, or hamsters have been widely used for behavioral and molecular studies, giving many significant advances in the understanding of the mammalian circadian system.

The effects of nighttime light exposure (NLE) on physiology have also been studied using classical nocturnal rodents (e.g., rats, mice and hamsters) [32]. Nonetheless, it is not evident at what level these might be useful as the best models for translational studies in human chronobiology.

**Figure 1:**
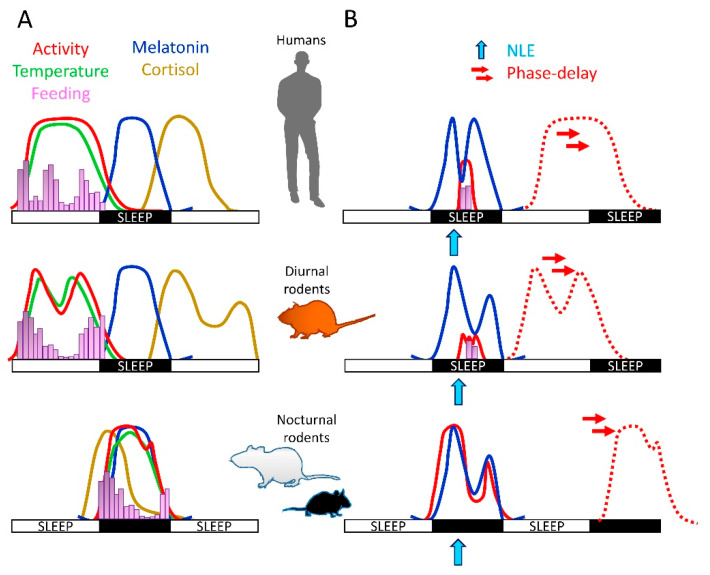
(**A**) Daily rhythms of physiology in diurnal (humans and rodents) and nocturnal (mice, rats) mammals are entrained to the light-dark (LD) cycle. Except for the daily rhythm of melatonin (MEL) that peaks at night in both diurnal and nocturnal mammals, other physiological rhythms show opposed phases between diurnal and nocturnal species. (**B**) Nighttime light exposure (NLE; blue arrow) affects in a similar manner behavioral and physiological rhythms in both humans and diurnal rodents (suppression of MEL; triggers arousal and eating; phase-shifts of activity rhythms). In nocturnal rodents, NLE also phases delays (double red arrows) rhythms of locomotor activity (dotted red lines) and suppresses MEL. However, different to diurnal species, NLE suppress locomotor activity and does not induce feeding. Therefore, the use of diurnal rodent species needs to be considered for translational studies and for the understanding of the negative consequences of light at night in human physiology.

Nocturnal rodents, unlike humans, sleep during the day and behave at night; although the hormone MEL is released in a circadian manner with a peak of release at night in both diurnal and nocturnal species [33,34,35] (Figure 1). Moreover, whereas NLE provokes awakening in humans, it triggers sleep in nocturnal rodents; thus, the use of nocturnal rodents for translational studies remains limited.

Some of the negative effects of NLE on physiology are in part due to the sleep deprivation and MEL suppression induced by light [36]. Nevertheless, in many studies using nocturnal mice, MEL production is deficient (C57BL6 strain). This highlights an important factor, different between diurnal vs. nocturnal species, to consider when we try to understand the effects of NLE on human physiology.

For some years now, some diurnal rodent models have been available for the study of the neuronal and molecular mechanisms of the circadian system, which can complement and reinforce existing knowledge in chronobiology in nocturnal rodents [37].

Circadian expression of clock genes, as well as electrical and metabolic activity in the SCN, is similar between nocturnal and diurnal species [38,39,40,41]. In addition, photic phase resetting of circadian rhythms (i.e., locomotor activity rhythms) and gene expression (*Per*) in the SCN induced by light are also similar between day- and night-active species [38,39].

Notwithstanding, clock gene expression in peripheral clocks and extra-SCN circadian oscillators are phase-opposite between day vs. night species [42,43,44]. In the brain, *Per* gene expression in structures such as the cortex, striatum, or hippocampus exhibits high amplitude and peak at day in the ground squirrel and the diurnal rodent *Octodon degu*, and at dusk in nocturnal species (e.g., rats, mice, hamsters) [42,43,44,45].

In addition, a recent study revealed daily rhythms of gene expression in central and peripheral organs in a diurnal primate, the *Papio anubis* (baboon); these rhythmic profiles showed a different phase from that of nocturnal species [46].

In another study in diurnal non-human primates, it was reported that the knockdown of the clock gene BMAL1 induces circadian (e.g., loss of hormonal rhythms) and sleep alterations, which were potentiated when primates were exposed to constant light conditions [47]. Furthermore, BMAL1-KO primates showed anxiety and depressive-like behaviors [47]. Interestingly, in nocturnal mice the specific *Bmal1* gene deletion in the SCN causes anxiety-like behavior [48]. Therefore, these comparative results between diurnal and nocturnal species arise two important points: (1) the role of clock genes in circadian and non-circadian behavior in nocturnal and diurnal species and (2) the possible use of diurnal species for translational studies in circadian biology in physiological and pathophysiological conditions.

In addition to the phase differences of peripheral clocks between diurnal and nocturnal mammals, non-circadian effects of light have been reported to be different between day- vs. night-active rodents, for example, the masking effects of light in locomotor activity are opposite between mice (nocturnal) and the diurnal rodent *Arvicanthis niloticus* (diurnal). Whereas light stimulation at night in nocturnal rodents induces a significant suppression of locomotor activity (negative masking), in diurnal rodents it induces an increase of locomotion and arousal (positive masking) [49,50] (Figure 1).

At the cellular level, light at night induces the expression of the protein c-FOS in the brain of the diurnal grass rat *Arvicanthis niloticus* and the nocturnal mouse. However, except for the SCN, a brief light stimulation at night leads in different c-FOS activity in specific brain areas between species [49]. Among those areas with opposite c-FOS responses to light, the LHb; in the diurnal rodent *Arvicanthis,* but not in mice, light at night triggers a significant increase of the protein c-FOS in the LHb [49]. In addition, a striking anatomical difference in the LHb between nocturnal and diurnal species was recently observed. In this study, authors reported that the glutamic acid decarboxylase (GAD), the essential enzyme in the formation of the neurotransmitter GABA, is present in the LHb of the diurnal Nile grass rat but not in the nocturnal rat [51]. The functional role of GAD in the LHb of diurnal *Arvicanthis* remains to be determined.

In humans, light also affects the activity of the habenula (Hb). A human’s fMRI study revealed that the Hb is sensitive to light changes in healthy volunteers. Hb activity is reduced when subjects were exposed to a change in luminance. Moreover, these changes are circadian-dependent, with a much more significant decrease of the Hb activity by light at morning than afternoon [52]. Whether luminescence increases Hb activity in humans at night as in diurnal rodents (c-FOS expression) has not been yet reported [49]. Notwithstanding, these findings give important evidence on how light affects brain structures (beyond the SCN) in humans, and how this can lead in changes in behavior (e.g., mood, cognition, etc.).

The LHb is a key structure to highlight due to its circadian properties and role in the regulation of behavior [53,54]. The LHb shows a self-sustained circadian profile with a high amplitude and peak of electrical activity and clock gene expression at daytime in nocturnal rodents [55,56,57].

If in diurnal species the LHb shows a daily or circadian rhythm of activity (i.e., electrical, gene expression, metabolic) similar to that observed in nocturnal rodents is not known. However, according to the previous results in which light at day decreases activity of the human Hb [52], and increases of c-FOS expression at night in diurnal rodents [49], it is possible that the daily activity of the diurnal LHb peaks at daytime. This is an important issue to point out when considering the LHb as part of the circuit that mediates the effects of light (light therapy) on depressive-like behaviors [25,58].

In short, because behavioral, cellular, and molecular responses to light are similar between humans and diurnal rodents, but opposite to nocturnal rodents, it would be interesting and relevant to propose the use of diurnal animal models (in addition to the nocturnals) to study the mechanisms underlying the effects of light (benefit or negative) on human physiology (Figure 1).

## 5. Nighttime Light Effects on Sleep and Circadian Biology of Diurnal Mammals

Currently, people worldwide are constantly exposed to nocturnal light due, in part, to the high availability and sometimes the “necessity” of using electronic devices emitting blue light (e.g., smartphones, tablets, computers). The excessive use of these devices at night leads to alterations in physiology. Amongst the negative consequences induced by NLE are the alterations of the sleep-wake cycle, which in a long term may lead in cognitive and mood deficits [59,60,61]. Under laboratory-controlled conditions, NLE, by the use of electronic tablets, induces alterations in sleep, such as a reduction of rapid eye movement (REM) sleep and an increase of alertness and of the latency to initiate sleep [60,61,62,63].

Previous studies have reported significant correlations between the exposure to indoor room illumination at night and sleep quality (i.e., latency to initiate sleep, sleep efficiency, wake after sleep onset, reduced total sleep time) in healthy young volunteers and elderlies. In healthy young male and female volunteers, the exposure to room light at nighttime affects REM sleep (increases awaking after sleep onset), leading to a reduction of total time of sleep and poor sleep quality [64,65]. Moreover, room light exposure at night induces a significant suppression of the production of MEL, and a delay of the daily rhythm of secretion [36].

In elderly individuals exposed to light at night under home setting conditions, similar effects were observed on sleep (i.e., increase of the latency to initiate sleep) [66]. Furthermore, in subjects suffering from bipolar disorder, a significant correlation between the intensity of light exposure at night and the presence of manic symptoms and altered sleep (e.g., latency to initiate sleep and episodes of waking during sleep) was reported [67,68].

Many studies on the effects of NLE on physiology have been conducted in laboratory and in home setting conditions. However, other studies have reported the negative consequences of NLE outdoors on physiology (light pollution). Adolescents, who spend important time exposed to light at night using electronic devices, are also the most exposed to outdoors light. In fact, nighttime outdoor light exposure is positively associated with a later bedtime, mood alterations, and anxiety in adolescents [69].

Together, these studies have significantly correlated indoor and outdoor NLE to sleep, hormonal, and circadian perturbations in humans. What are the possible brain mechanisms that underlie these effects?

Beyond the entrainment of the circadian system, light reaches the brain to activate, inhibit or modulate its activity and then behavior. ipRGCs project to other extra-SCN brain sites to control and regulate sleep, mood, cognition, and reward [4]. Within the extra-SCN structures targeted by ipRGCs are the principal areas implicated in the regulation of sleep such as the ventrolateral preoptic area (VLPO; sleep-promoting neurons) and the lateral hypothalamus (LH; wake-promoting neurons) [23,70]. Thus, sleep can be affected directly by light and independently of the SCN clock.

In nocturnal mice, light affects sleep in a wavelength-dependent manner [71]. Whereas blue-rich light (470 nm) induces arousal and delays the sleep onset, green light (530 nm) induces a rapid sleep onset. The opposite effects of blue vs. green light in arousal and sleep, respectively, lead in a differential increase of the cellular marker c-FOS in the SCN (by blue light) and the VLPO (green light). These data confirm the role of the VLPO in sleep promotion, and give new insights on the brain pathways playing a role in the regulation of sleep by light [71]. Nonetheless, this study was conducted in nocturnal mice; thus, light (blue or green) exposure at night was at the activity phase [71]. Therefore, it will be interesting to evaluate the effects of blue and green light on sleep using diurnal rodents.

In fact, spontaneous c-FOS activity in sleep and wake promoting brain areas is opposite between nocturnal rats and the diurnal rodent *Arvicanthis niloticus*. The daily rhythm of c-FOS activity in the sleep-promoting VLPO shows a higher amplitude and peak at the light onset in rats (rest period) [72], and at night (inactive period) in *Arvicanthis* [73]. These results raise the question whether light at night (blue, green) in diurnal species, or even in humans, has similar effects on sleep regulation as in nocturnal species.

Sleep has been well studied in nocturnal rodents such as rats and mice [74]. Importantly, there are interesting data on the characterization of sleep physiology in diurnal chipmunks and ground squirrels [75], and in the diurnal rodents *Octodon degus* and *Arvicanthis ansorgei* [76,77]. In the diurnal *Arvicanthis ansorgei* it has been reported that both NREM and REM sleep occur at dark period, thus, opposite to that observed in nocturnal rodents (i.e., rats, mice) [77]. Furthermore, as in humans, light stimulation at night decreases pineal MEL levels and induces arousal in the *Arvicanthis ansorgei* [33,77] (Figure 1). Hence, it is conceivable that NLE in diurnal rodents induces sleep alterations similar to those observed in humans; an interesting hypothesis for translational studies on the effects of light at night in sleep homeostasis.

## 6. Nighttime Light Effects on Mood of Diurnal Mammals

Sleep disturbances are often followed by mood disorders (e.g., depression). Patients with major depressive disorder (MDD) exhibit alterations of the rhythms of hormone release (e.g., melatonin) and of the sleep-wake cycles [78,79]. One of the possible causes that alter circadian rhythms in MDD is a poor entrainment to the day-night cycle [80].

Seasonal affective disorder (SAD) has also been related to a poor light entrainment (low light intensity exposure). People with SAD show anxiety and depressive symptoms principally in winter when natural day light intensity and duration are low and short, respectively [81]. Therefore, daytime bright light exposure is a good therapeutic option to treat MDD, SAD, and other psychiatric disorders in part due to the synchronization of the circadian system [81,82,83].

Despite some studies in humans showing brain changes induced by seasons, the central mechanisms implicated in SAD and in the therapeutic effects of bright light exposure are not totally known, although the brain monoaminergic (e.g., serotonin, dopamine) activity could be strongly implicated [84,85,86,87].

SAD rat and mice (nocturnal) models show alterations in both the circadian and monoaminergic systems [88]. Moreover, some previous studies have reported possible brain mechanisms of SAD using diurnal rodents as models.

Diurnal *Arvicanthis niloticus* exposed to low levels of light at daytime develop depressive and anxiety-like behavior with elevated levels of corticosterone (stress response), and morphological neural changes in the hippocampus [89]. Furthermore, these LD housing conditions (low light intensity or shorter day-length) lead to a deficient serotonergic signaling, without affecting daily rhythms of locomotion or SCN clock proteins expression. This suggests a SCN-independent implication of the brain monoaminergic pathways on the effects of daytime low intensity light exposure in behavior of diurnal rodents [90,91].

In another study, the day-night variations of the dopamine content in the forebrain are altered in the diurnal grass rat *Arvicanthis ansorgei* exposed to winter-like photoperiod conditions. Interestingly, these effects are reversed when animals are treated with daily light (1 h) stimulation [92]. In similar diurnal species (*Arvicanthis niloticus*), light exposure at the circadian day increases c-FOS expression in hypothalamic orexin neurons and in serotonergic cells from the dorsal raphe nucleus [93]. Overall, these results indicate that mood-related behavior in diurnal rodent species is sensitivity to changes in light intensity and duration. Moreover, these studies support the hypothesis on the direct effects of daytime light exposure on the monoaminergic system (e.g., serotonin, dopamine) to improve mood-related behaviors.

In spite of the therapeutic effects of light at daytime in mood in humans (and diurnal rodents), nighttime light exposure may have important consequences in mood in both humans and animal models.

Adolescents use light emitting devices at night, in an excessive manner, allowing a considerable increase of NLE before bedtime. This condition has been considered as a risk factor for the development of mood disorders. Teenagers (12–17 year old) who use smartphones in bed before sleep showed a reduced sleep duration and depressive symptoms [61]. The negative effects of NLE appear to be more pronounced in adolescents due to their transparent lenses that permit a higher sensitive to light. Indeed, adolescents exposed to light at night show a strong suppression of MEL, larger phase-shifts of the circadian system, and poor sleep [94].

The effects of light at night on circadian physiology (phase changes) are intensity, time, duration, and wavelength dependent [95]. Thus, it is possible that the effects of light at night in non-circadian biology (e.g., mood, cognition) are also dependent on these variables.

For example, in elderly people, light exposure at night in home settings (indoor light) is significantly associated with depressive symptoms, being higher in individuals with the exposure to intensities ≥5 lux. This correlation was independent of other factors such as sleep habits, physical activity, or even light exposure at daytime. Furthermore, depressive symptoms disappear when subjects are exposed to constant darkness at nighttime [96,97].

Recent studies evaluated the possible correlation between outdoor light exposure at night and the presence of depressive symptoms. For that purpose, researchers selected people from a Dutch (18 to 65 years old) and a Korean population (20–59 years old), and using a satellite measure of exposure to nocturnal illumination outdoors, they reported a positive correlation between depressive symptoms and the levels of light exposure [98,99].

In animal models, few studies have reported the effects of light at night in mood-related behaviors using diurnal rodents. Diurnal rodents *Arvicanthis niloticus* exposed to a LD cycle with dim light at night phase (5 lux) showed anhedonia (decrease of sucrose preference) and behavioral despair (more immobility in the forced swim test), with a reduction of the dendritic length in neurons from the hippocampus, but without alterations of the daily rhythms of locomotion [100].

On the other hand, nighttime light exposure in diurnal grass rats produces an increased immune response and elevated corticosterone concentrations [101]. This study suggests the possibility that light at night may act as a long-term stressor inducing alterations in the glucocorticoid signaling and the immune response, which may lead to the development of mood alterations [101].

Since light at night impacts extra-SCN brain regions differentially between nocturnal vs. diurnal rodents [49], and some of them are importantly implicated in the regulation of mood (LHb) [102], research on the mechanisms underlying the effects of NLE on mood-related behavior in diurnal species remains to be explored.

## 7. Nighttime Light Effects on Eating and Metabolism of Diurnal Mammals

In industrialized countries, in addition to the negative effects of light pollution, society faces the negative consequences of unhealthy overeating; both conditions have been correlated with the development of metabolic diseases such as obesity and diabetes, two major global public health problems [103]. Even worse, the exposure to light and eating at night might enhance the negative effects on human health. Night workers are a clear example of people exposed to light at night and prone to developing overeating and obesity; thus, this is an important group in society strongly affected by NLE [104].

Recent studies have evidenced important effects of light at night in metabolism and feeding in both humans and animal models. For example, night blue-enriched light exposure in healthy adults induces higher values of blood glucose, accompanied of a reduction in subjective sleep [105].

In the elderly, NLE has been associated with body weight increase and lipid dysregulation (dyslipidemia) in both male and female volunteers [106]. Interestingly, in this study NLE-exposed subjects showed significantly higher waist circumference (an indirect measurement of body fatness). These effects were independent of other physiological factors such as age, sleep duration, or MEL levels. Therefore, although sleep loss has been associated with an increase in food intake, hunger, and body weight, in this study the effects of NLE in body weight could be related to other mechanisms independent of sleep deprivation [66].

In a subsequent study in elderly volunteers, authors reported that the effects of NLE on body weight are time-dependent; the correlation between NLE and the increase of body mass index (BMI) and the ratio of waist circumference is more significant when light occurs at late vs. early night [107]. Moreover, in some elderly participants, there was a significant association between NLE and the increase on the incidence to develop diabetes [108]. On the other hand, the exposure to long periods of darkness at night and to light at daytime was associated to a lower BMI and to a reduced ratio of waist circumference [107]. Similarly, in a large cohort study in UK women, BMI and waist circumference increased in subjects exposed to light at night [109].

In another recent longitudinal study, a significant association between body weight increase and light exposure at night in women while sleeping was identified [110]. In fact, authors followed, for more than five years, a group of women that had the habit of sleeping with the television or lights on in their room. Under these conditions, women with a greater light exposure to light at night showed a significant and positive correlation with being overweight and the risk of developing obesity [110].

Many of the studies of the effects of NLE on metabolism have been reported in adults or the elderly. However, it would be interesting to evaluate if these effects are also observed in a youngest population. A late chronotype has been associated with an increase in caloric intake and obesity [111,112]. Teenagers and young adults show a night chronotype and are often exposed to higher intensities of light at night. Furthermore, the circadian system of adolescents is more sensitive to light [94]. Therefore, the effects of NLE on body weight gain remain to be determined in teenagers and young adults.

NLE also affects eating behavior and metabolism. In healthy young individuals, a brief light exposure at night alters hormonal and metabolic profiles. In the metabolic response, healthy people exposed to bright light showed post-meal higher glucose and insulin levels compared to dim-light exposed subjects [113]. Moreover, in another study, using a similar protocol of light exposure, it was observed that healthy subjects exposed to bright light at night report more appetite, hunger and desire to eat (Figure 1) [114].

Several studies on the effects of NLE in glucose and lipid metabolism, body weight, and food intake have been conducted in nocturnal rodents such as rats and mice [32]. Interestingly, a recent study using diurnal species describes new data on the effects of NLE in feeding and metabolism.

In the diurnal rodent *Arvicanthis ansorgei,* a single exposure to blue light at the beginning of the night (rest period) dysregulates glucose metabolism and induces feeding. In fact, animals exposed to 1 h of blue light and to a glucose tolerance test showed glucose intolerance and a reduced insulin secretion, possibly by a decreased beta cell activity [115]. No effects were observed on leptin concentrations by NLE. Similarly, in humans, bright light exposure does not affect plasma leptin concentrations [116]. Leptin is a hormone that stimulates satiety and shows a circadian rhythm controlled by the SCN in both rodents and humans [117,118]. Since light at night resets the SCN, thus, changes in leptin by NLE may possibly be observed in the timing (phase) of the circadian rhythm.

Interestingly, in nocturnal rats, green light, but not blue light exposure at night, leads in similar glucose intolerance as in diurnal *Arvicanthis* exposed to blue light [119]. These results suggest that the effects of NLE on glucose metabolism between diurnal and nocturnal rodents are wavelength-dependent.

In diurnal *Arvicanthis*, the increase in food intake by light exposure was diet- and gender-specific. In *Arvicanthis* fed with a highly palatable meal containing fat and sugar, blue NLE triggers sucrose intake in male, but not female, animals (Figure 1). These results highlight the relevance of melanopsin cells, which are more sensitive to low wavelength light (blue-rich), on the effects of NLE on metabolism and food intake.

Notably, in nocturnal rats, different to diurnal *Arvicanthis*, the exposure to dim-light at night decreases nighttime feeding and energy expenditure [120]. In addition, green and blue light exposure at the late night reduces food intake in rats [121].

These last data are important to consider for further translational studies, not only for the difference between the nocturnal phenotype of rats and the diurnal nature of *Arvicanthis*, but also for their retinal differences (sensitivity, morphology) [122]. Furthermore, the opposite effects of NLE in feeding behavior between diurnal and nocturnal species should be taken into consideration when attempting to explain the effects of light at night in glucose metabolism between day- and night-active rodents.

Therefore, due the similar effects of NLE on feeding between the diurnal rodents and humans, for translational studies it will interesting to use this animal species, despite the comparable findings of NLE effects on glucose metabolism in nocturnal rats and diurnal *Arvicanthis*.

What are the mechanisms underlying the effects of light on eating and metabolism? This question remains to be answered, but different hypotheses have been proposed. One of these is the misalignment among the central pacemaker (SCN) and peripheral circadian clocks regulating metabolism (e.g., pancreas, liver).

Furthermore, NLE may affect metabolism through the SCN clock and the autonomous nervous system, by which light can contact peripheral organs (e.g., liver, pancreas, adrenals) [123,124].

On the other hand, due to the direct effects of light in other brain substrates regulating energy balance and feeding (e.g., hypothalamus, limbic substrates), NLE may directly reach these structures to induce eating or alter metabolism [23]. However, these are still hypotheses that remain to be confirmed.

Another mechanism by which NLE alters metabolism might be through MEL signaling. MEL release at night is suppressed by light in an intensity-dependent manner. MEL has an important role in metabolism acting in peripheral tissues, such as the liver or pancreas for the regulation of glucose and insulin, respectively [125]. However, this mechanism cannot be considered to explain the effects of NLE on metabolism of nocturnal MEL-deficient mice. Therefore, studies using MEL-proficient diurnal rodents, with similar sleep-wake cycles as humans, would support the possible role of MEL in the effects of NLE on metabolism.

## 8. Conclusions

Currently, fundamental research in natural sciences uses principally nocturnal rodents such as rats and mice giving an enormous advance in the understanding of physiology and behavior. In some fields of biological research, the nocturnal nature of animal models seems to be not relevant. However, in chronobiology this might be an important issue to look at, mainly when we try to understand the human circadian timing system in physiological and pathophysiological conditions. The proposition of diurnal rodents is limited, but the few studies using these give important and new data to consider in the study of the effects of light, or other time cues (e.g., food, exercise) [126] on the circadian system. Furthermore, recent data in diurnal primates (closer to human physiology) support the relevance of the use diurnal species for translational studies [37,46,47]. This will increase and reinforce the very important current knowledge of the circadian system in nocturnal rodents. The use of diurnal models may provide an additional and valuable understanding of human circadian timekeeping in health and disease.

## Data Availability

Not applicable.
